# Eyelid Dysfunction in Neurodegenerative, Neurogenetic, and Neurometabolic Disease

**DOI:** 10.3389/fneur.2017.00329

**Published:** 2017-07-18

**Authors:** Ali G. Hamedani, Daniel R. Gold

**Affiliations:** ^1^Department of Neurology, Hospital of the University of Pennsylvania, Philadelphia, PA, United States; ^2^Department of Neurology, Johns Hopkins Hospital, Baltimore, MD, United States; ^3^Department of Ophthalmology, Johns Hopkins Hospital, Baltimore, MD, United States; ^4^Department of Neurosurgery, Johns Hopkins Hospital, Baltimore, MD, United States; ^5^Department of Otolaryngology – Head and Neck Surgery, Johns Hopkins Hospital, Baltimore, MD, United States

**Keywords:** eyelid, neurodegenerative diseases, neurogenetic, blinking, blepharospasm, Parkinson, movement disorders

## Abstract

Eye movement abnormalities are among the earliest clinical manifestations of inherited and acquired neurodegenerative diseases and play an integral role in their diagnosis. Eyelid movement is neuroanatomically linked to eye movement, and thus eyelid dysfunction can also be a distinguishing feature of neurodegenerative disease and complements eye movement abnormalities in helping us to understand their pathophysiology. In this review, we summarize the various eyelid abnormalities that can occur in neurodegenerative, neurogenetic, and neurometabolic diseases. We discuss eyelid disorders, such as ptosis, eyelid retraction, abnormal spontaneous and reflexive blinking, blepharospasm, and eyelid apraxia in the context of the neuroanatomic pathways that are affected. We also review the literature regarding the prevalence of eyelid abnormalities in different neurologic diseases as well as treatment strategies (Table 1).

## Ptosis

### Overview of Eyelid Elevation

During wakefulness, the muscles of eyelid elevation are tonically activated to maintain eye opening against the passive tendency of the eyelids to close, and the muscles of eyelid closure are silent except during blinks. Thus, ptosis is by definition a problem of reduced eyelid elevation rather than excess eyelid depression.

**Table 1:** Summary of eyelid disorder mechanisms, associations, and treatments in neurodegenerative and neurogenetic disease.

Eyelid disorder	Mechanism(s)	Associated conditions	Treatment(s)
Ptosis	LPS weakness	CPEO spectrum, myotonic dystrophy, OPMD, congenital myasthenic syndromes, SCA28	Eyelid taping and crutches, surgical myectomy or frontalis suspension

	LPS fibrosis, dysgenesis, or dehiscence	Congenital ptosis, CFEOM	

	Oculosympathetic dysfunction	Congenital disorders of neurotransmitter synthesis	

Eyelid retraction	Dissociation between eye and eyelid position due to impaired supranuclear control of the M-group resulting in excess CCN activity	PSP, SCA3	Ocular lubrication to prevent exposure keratopathy due to increased corneal exposure

Decreased blinking	Reduced nigrocollicular pathway activity resulting in greater inhibition of spontaneous blinking	Parkinsonism (PSP > PD)	Ocular lubrication; dopaminergic therapy to treat underlying movement disorder

Increased blinking	Increased nigrocollicular pathway activity resulting in reduced inhibition of spontaneous blinking	Hyperdopaminergic disorders (e.g., HD)	Dopaminergic blockade or reduction to treat underlying movement disorder

Blepharospasm	Blink reflex hyperexcitability	Idiopathic, with or without eyelid apraxia; Meige syndrome and other dystonias; parkinsonism (PSP >> PD); SCAs	Botulinum toxin, polarized lenses, surgical myectomy (especially if comorbid eyelid apraxia) or DBS

Eyelid apraxia	Excess supranuclear LPS inhibition with or without pretarsal OO activation	Idiopathic, with or without blepharospasm; parkinsonism (PSP > MSA > PD); ALS	Botulinum toxin (specifically to pretarsal OO), eyelid crutches or goggles, trial of levodopa or other medications, rarely surgical myectomy frontalis suspension (especially if comorbid blepharospasm)

*LPS, levator palpebrae superioris; CPEO, chronic progressive external ophthalmoplegia; OPMD, oculopharyngeal muscular dystrophy; CFEOM, congenital fibrosis of the extraocular muscles; SCA, spinocerebellar ataxia; CCN, central caudal nucleus; PSP, progressive supranuclear palsy; SC, superior colliculus; PD, Parkinson’s disease; HD, Huntington’s disease; DBS, deep brain stimulation; OO, orbicularis oculi; MSA, multiple systems atrophy; ALS, amyotrophic lateral sclerosis*.

**Figure 1:**
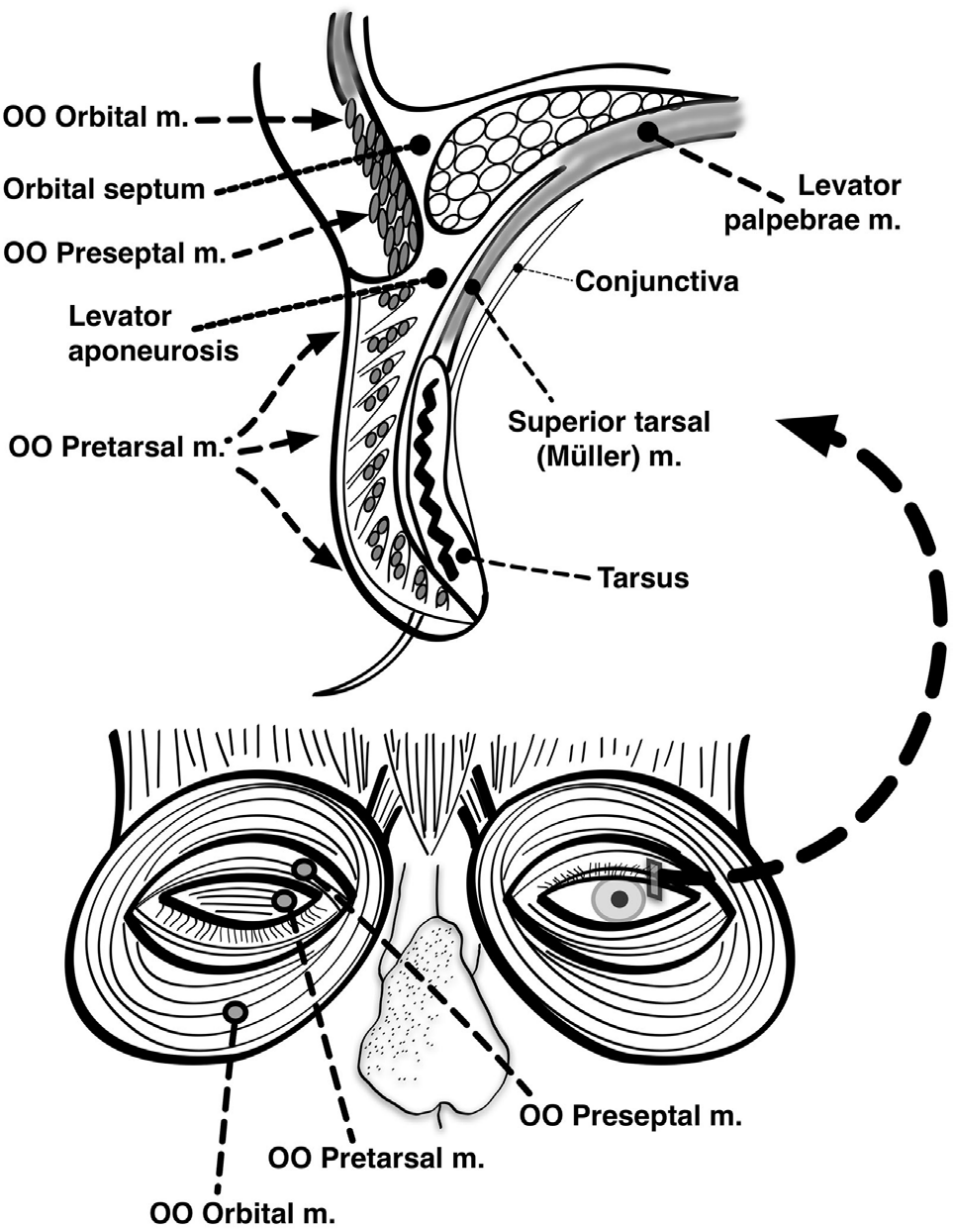
Anatomy of the eyelids. Seen here are the major muscles of eyelid opening and closure. The levator palpebrae, which is innervated by the oculomotor nerve, inserts on the tarsus *via* the levator aponeurosis and directly on the skin of the upper eyelid. The superior tarsal muscle (also known as Muller’s muscle, which is innervated by oculosympathetic fibers) originates from the levator aponeurosis and inserts on the tarsus. The orbicularis oculi (OO) is innervated by the facial nerve. It is made up of two portions: one contained within the eyelid itself (palpebral portion) and one located outside the eyelid surrounding the orbit (orbital portion). The palpebral portion of the OO can be further subdivided into preseptal and pretarsal components based on its anatomic location relative to the tarsus (Modified with permission from (154), Figure 24.5).

The primary muscle of upper eyelid elevation is the levator palpebrae superioris (LPS), which is innervated by the oculomotor nerve. It originates from the lesser wing of the sphenoid bone at the orbital apex, courses through the orbit superior to the superior rectus (SR) muscle, and inserts on the superior tarsal plate as well as directly on the skin of the upper eyelid, forming the lid crease. A secondary muscle (the superior tarsal muscle, also known as Müller’s muscle) originates from the distal aponeurosis of the LPS and inserts on the superior tarsal plate as well. In the lower eyelid, the inferior tarsal muscle analogously inserts on the inferior tarsal plate. The tarsal muscles are both innervated by oculosympathetic nerve fibers arising from the superior cervical ganglion. The frontalis and other facial nerve-innervated muscles can indirectly affect eyelid position as well (Figure 1).

Several clinical measurements can aid in the localization and assessment of ptosis (1):

1. Palpebral fissure height, which is the distance between the upper and lower eyelids at rest in primary gaze and is normally at least 10 mm. This can be subdivided into the margin reflex distance (MRD) 1, which is the distance between the corneal light reflex and the upper eyelid margin, and the MRD 2, which is the distance between the corneal light reflex and the lower eyelid margin.
2. Lid crease height, which is the distance between the lid crease and upper eyelid margin as measured in downgaze and is normally less than 10 mm. The most common cause of ptosis with heightened lid crease is the levator dehiscence–disinsertion syndrome (see below).
3. Eyelid excursion or levator function, which is the difference in position of the upper eyelid margin in upgaze compared to downgaze and is normally greater than or equal to 12 mm.

Of these parameters, perhaps the most useful is eyelid excursion, as it differentiates ptosis with reduced levator function from ptosis with preserved levator function. Ptosis with reduced levator function implies a lesion of the LPS or its motor control.

### Ptosis due to Levator Weakness

Because it is very rich in mitochondria (2), the LPS is preferentially affected in mitochondrial myopathies. This is illustrated by the prominent ptosis that accompanies the *chronic progressive external ophthalmoplegia (CPEO)* phenotype, which can occur in isolation or in association with other mitochondrial syndromes, such as the Kearns–Sayre syndrome, sensory ataxic neuropathy with dysarthria and ophthalmoplegia, Leigh syndrome, and mitochondrial neurogastrointestinal encephalopathy among others (3). CPEO can be caused by either mitochondrial or nuclear DNA mutations (4). When the mitochondrial genome is affected by large deletions, rearrangements, or point mutations involving genes encoding for tRNA synthetases, mutations are typically somatic rather than germline, resulting in a sporadic rather than maternal pattern of inheritance. Mutations in nuclear genes can also cause CPEO and may be inherited in an autosomal dominant or recessive manner. To further complicate matters, many of these nuclear genes (e.g., OPA1) are responsible for mitochondrial homeostasis, and thus patients with inherited nuclear DNA mutations can acquire somatic mitochondrial DNA mutations over time (5). This may account for some of the phenotypic variability of these diseases. Ptosis and ophthalmoparesis may also be seen in some of the autosomal dominant spinocerebellar ataxias (SCAs) (6), particularly SCA28, which is caused by mutations in the *AFG3L2* gene (7). Interestingly, SCA28 patients have been shown to accumulate mitochondrial DNA mutations (8), suggesting a mechanism for which ptosis might appear in an otherwise purely cerebellar and pyramidal syndrome. A CPEO-like syndrome accompanied by symmetric parkinsonism has been reported in families with *c10orf2* (Twinkle) and *POLG1* mutations (9), and ptosis has also occurred in cases of early-onset Parkinson’s disease (PD) due to *PARK2* mutations (10). Otherwise, ptosis is not a typical manifestation of PD or other acquired neurodegenerative disorders.

Most other inherited myopathies spare the eyelids and extraocular musculature. Notable exceptions include *oculopharyngeal muscular dystrophy (OPMD)* and *myotonic dystrophy*. The pathologic hallmark of OPMD on muscle biopsy is filamentous intranuclear inclusions composed of the misfolded polyalanine expanded *PABPN1* protein [which is similar to other trinucleotide repeat diseases such as Huntington’s disease (HD)], though aggregates of dysmorphic mitochondria have also been observed (11), which may be the mechanism by which the LPS is preferentially affected. A striking feature of myotonic dystrophy is that while it is an autosomal dominant disease, the phenotype is more severe when it is inherited maternally rather than paternally, and congenital presentations are seen exclusively in the children of affected mothers (12). These observations led to the hypothesis that the pathogenesis of myotonic dystrophy may be influenced by mitochondrial factors. However, several mitochondrial DNA sequencing studies have failed to detect any variants associated with phenotype severity (13). Congenital myasthenic syndromes also frequently cause ptosis. They are caused by mutations in a number of genes involved in neuromuscular transmission, both presynaptic and postsynaptic (14).

### Congenital Ptosis

Reduced levator function that is present at birth has a separate differential diagnosis, namely that of dysgenesis or fibrosis of the eyelid musculature. Isolated *congenital ptosis* is caused by dysgenesis and hypoplasia of the LPS, and typical clinical features include reduced or absent lid crease and lid lag in downgaze. Due to its shared embryologic origin with the SR, upgaze may also be affected (15). Most cases of congenital ptosis are unilateral and sporadic, but mutations in several genes have been identified in familial cases (16). Histologic examination reveals reduced muscle fiber number and fibrosis, leading many to initially suspect that the disease is primarily myopathic in pathogenesis (17). However, increasing recognition of aberrant reinnervation in these cases, including the Marcus-Gunn jaw winking phenomenon (whereby a ptotic eyelid retracts with lateral jaw movement as in sucking or chewing, suggesting innervation of the LPS by the trigeminal nerve), has led to dysinnervation-based theories, and some, therefore, group congenital ptosis with the congenital cranial dysinnervation disorders (CCDD) (18). *Congenital fibrosis of the extraocular muscles* is another CCDD that is thought to have a similar pathophysiology and produces a syndrome of ptosis and ophthalmoplegia that resembles CPEO except that it is present at birth and the ophthalmoplegia is restrictive in physiology (19). Of note, aberrant extraocular muscle innervation can produce secondary eyelid abnormalities without directly affecting the LPS. For example, in the Duane retraction syndrome, the palpebral fissure narrows during adduction not because of reduced eyelid opening or increased eyelid closure but because of simultaneous contraction of the medial and lateral recti due to dual innervation by the oculomotor nerve resulting in retraction of the globe into the orbit (20).

### Mechanical Ptosis

Ptosis with preserved levator function is usually caused by a defect of the aponeurotic insertion of the LPS onto the upper eyelid. *Levator dehiscence–disinsertion syndrome* is the most common cause of acquired ptosis in adulthood and occurs when the LPS loses its insertion site on the superior tarsal plate and then reinserts on a more proximal portion of the tarsal plate or eyelid skin. This results in an abnormally increased lid crease height with preserved eyelid excursion (21). It is commonly seen with advancing age but can be accelerated by eyelid manipulation during regular contact lens use, frequent rubbing of the eyes, botulinum injection of the orbicularis oculi (OO) for the treatment of blepharospasm (22), or ocular surgery. Ptosis with preserved levator function can be inherited in relative isolation as in the autosomal dominant blepharophimosis-ptosis-epicanthus inversus syndrome (BPES) or in the setting of other craniofacial abnormalities as in trisomy 13, Turner syndrome, Noonan syndrome, Cornelia de Lange syndrome, and many of the congenital arthrogryposes (23).

As discussed later in this review, the level of tonic LPS activity depends on vertical eye position. By contrast, the superior and inferior tarsal muscles remain equally active in all directions of gaze; they are not primary eyelid elevators but instead are modulated by level of arousal and sympathetic tone. Thus, a lesion of the tarsal muscles or their oculosympathetic innervation (as in Horner’s syndrome) results in mild to moderate ptosis with preserved levator function rather than the more severe ptosis with reduced levator function that is seen in true LPS weakness of neurogenic (e.g., oculomotor nerve palsy) or myogenic origin. The ptosis seen in disorders of neurotransmitter synthesis, such as tyrosine hydroxylase deficiency, aromatic l-amino acid decarboxylase deficiency, dopamine beta-hydroxylase deficiency, and brain dopamine–serotonin vesicular transport disease is thought to occur by this mechanism (24).

### Treatment of Ptosis

Symptomatic treatment of ptosis is generally reserved for cases where the degree of ptosis is so great as to obscure the visual field or cause cosmetic distress. Conservative measures include temporary taping of the upper eyelids or the use of crutches attached to eyeglasses. Surgical options include shortening of the LPS, resection of the superior tarsal muscle, and frontalis suspension to elevate the entire upper eyelid complex. Other than the usual risks of any surgical procedure, the primary risk associated with surgical treatment of ptosis is incomplete eyelid closure during normal blinking and sleep (lagophthalmos) causing exposure keratopathy. Surgery should, therefore, be approached with caution especially if the muscles of eyelid closure are also weak, as in CPEO and other myopathies.

## Eyelid Retraction

### Overview of Vertical Eye and Eyelid Position Coordination

**Figure 2:**
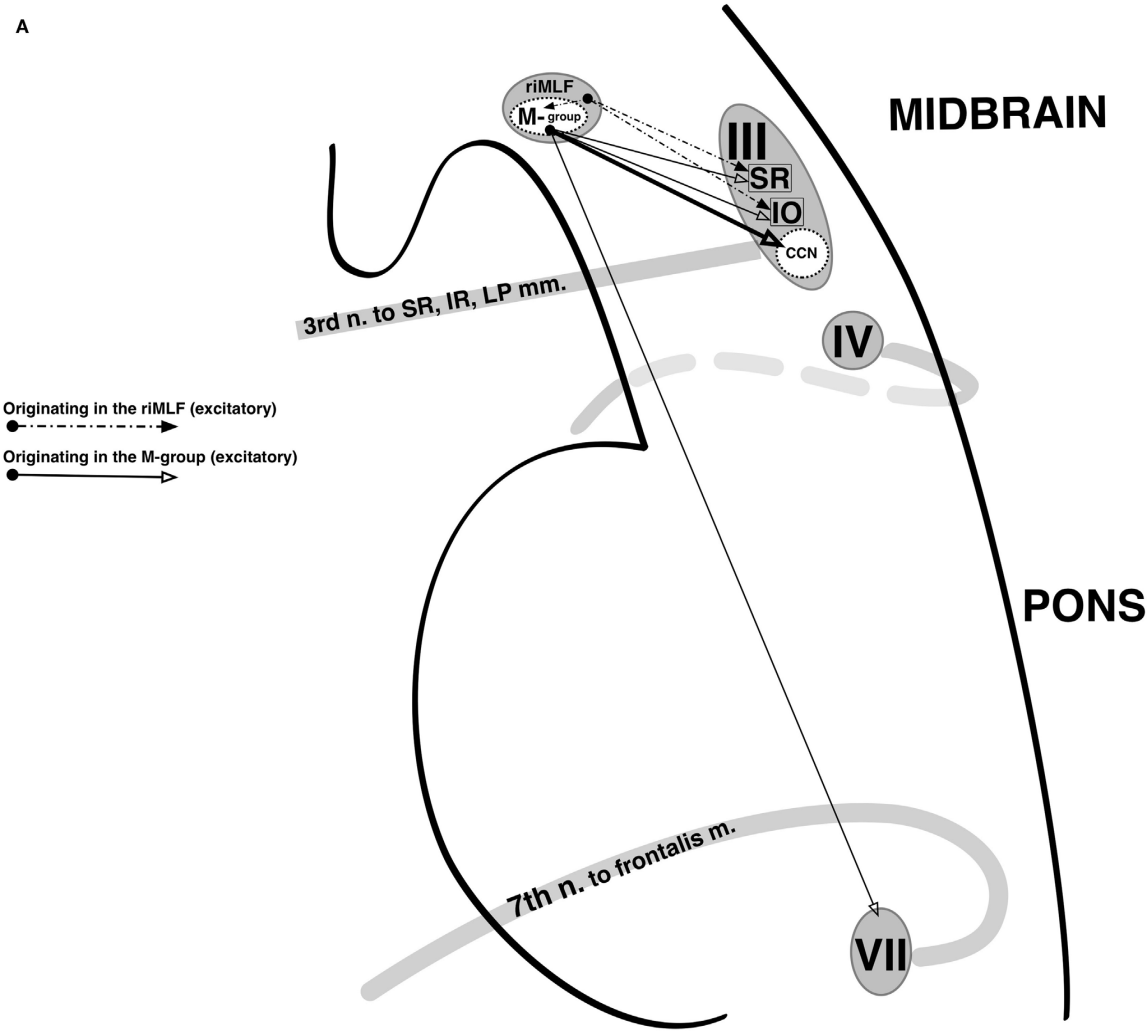

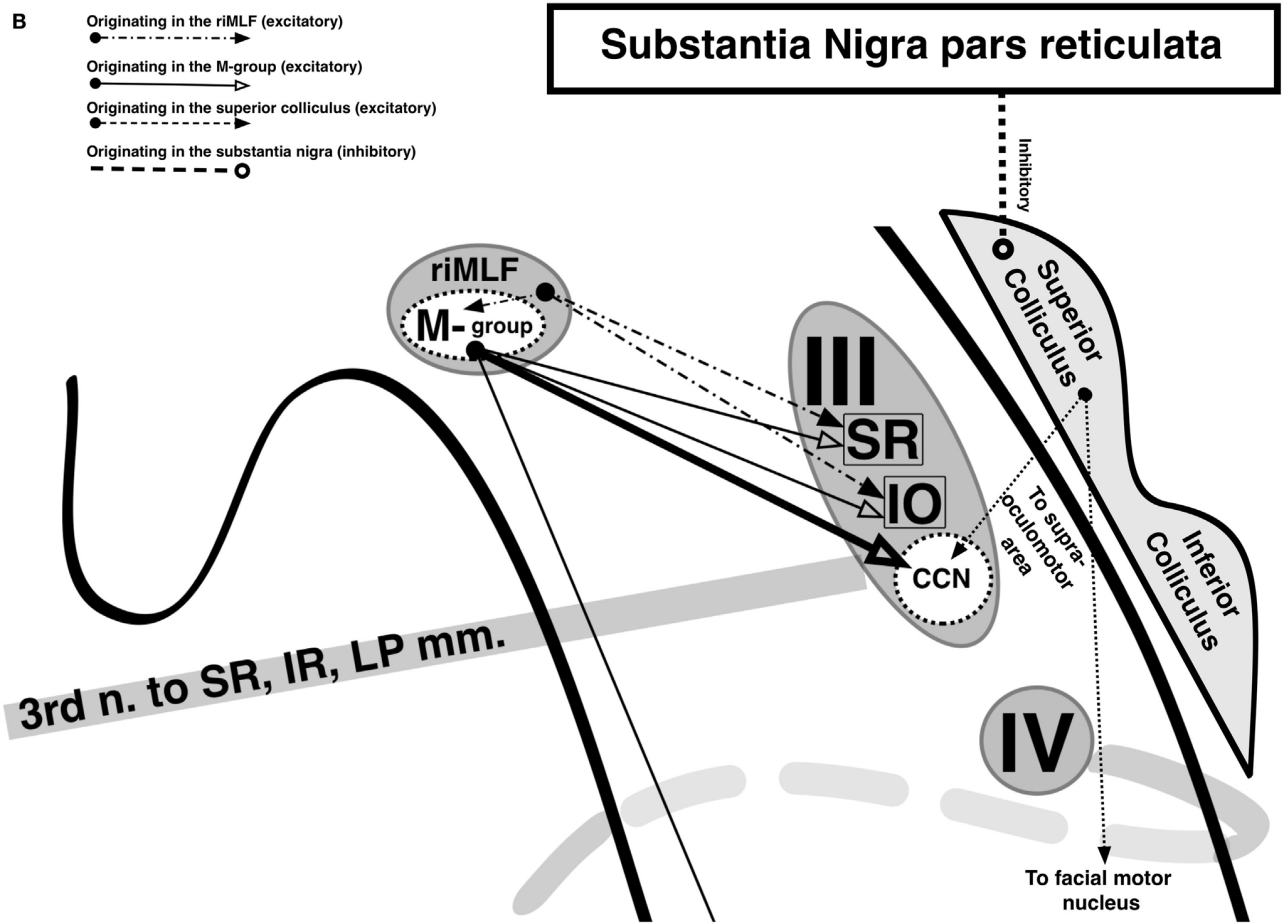
Supranuclear control of eyelid movement. **(A)** The central caudal nucleus (CCN) of the midbrain contributes fibers to both oculomotor nerves and innervates both levator palpebrae superioris (LPS). It maintains a tonic level of activity during eye opening that transiently increased with upward eye movements and decreases with downward eye movements. During a vertical saccade, the rostral interstitial nucleus of the median longitudinal fasciculus (riMLF) is activated, and it provides excitatory input into the superior rectus (SR) and inferior oblique (IO) subnuclei of the oculomotor nerve in order to elevate the eyes. In addition, the riMLF activates the nearby M-group. The M-group provides a small amount of reinforcing excitation to the SR and IO subnuclei, but its primary excitatory output is to the CCN, resulting in an increase in firing rate which produces eyelid elevation. The M-group also synapses on the facial nucleus, presumably to provide assistance from the frontalis in eyelid elevation when needed. The opposite occurs during downgaze. Eyelid retraction in midbrain dysfunction occurs due to M-group overstimulation (in an attempt to overcome an upgaze palsy) or underinhibition (from injury to the nearby interstitial nucleus of Cajal and nucleus of the posterior commissure). **(B)** During a blink, the LPS abruptly ceases firing and the orbicularis oculi (OO), which is innervated by the facial nerve, briefly contracts. This coordination of LPS and OO activity is thought to be mediated by the superior colliculus (SC). The SC projects to the supraoculomotor area directly overlying the CCN as well as to the facial nuclei and is inhibited by the pars reticulata of the substantia nigra (SNr). In parkinsonism, there is increased activity in the SNr, which results in greater inhibition of the SC and reduced spontaneous blinking. Not shown are afferents from the trigeminal nucleus and pretectum to the SC, which mediate reflexive blinking to corneal stimulation and bright light, respectively.

To maximize protection of the cornea and tear film while avoiding obscuration of the visual field, the eyelids normally elevate in upgaze and depress in downgaze with a velocity and gain that roughly matches that of the corresponding eye movement, be it a saccade or smooth pursuit (25). A single nucleus [the central caudal nucleus (CCN) of the midbrain] is shared by both the left and right oculomotor nucleus complexes and innervates both LPS muscles; eyelid elevation is, thus, yoked between the two eyes (26). In electrophysiologic studies of primates, the CCN has a basal firing rate in primary gaze, and in upward saccades, it experiences a burst of increased firing after which its basal rate resumes (25, 27, 28). Correspondingly, in downward saccades, it experiences a pause in firing, during which the eyelid passively depresses until it reaches its target level, at which point the basal firing rate resumes. Similar firing patterns have been recorded in the SR subnucleus during vertical saccades, suggesting shared supranuclear control with the CCN (28, 29). In primates, a population of neurons called the M-group lying adjacent to the rostral interstitial nucleus of the median longitudinal fasciculus (riMLF, which generates vertical and torsional saccades) sends projections to both the CCN and the oculomotor subnuclei responsible for supraduction (namely, the SR and inferior oblique) (30). It appears to receive excitatory input from the riMLF and superior colliculus (SC) during upgaze and inhibitory input from the interstitial nucleus of Cajal (iNC) and nucleus of the posterior commissure (nPC) during downgaze (31) (Figure 2).

### Eyelid Retraction in Midbrain Dysfunction

Disruption of these midbrain pathways is the mechanism by which central nervous system disease causes eyelid retraction. It is often accompanied by a vertical gaze palsy, as in the dorsal midbrain syndrome (also known as the *pretectal or Parinaud syndrome*, where eyelid retraction is referred to as Collier’s sign) (32) and *progressive supranuclear palsy (PSP)*. Eyelid retraction in these disorders reflects a dissociation between eye position and eyelid position such that the CCN is relatively overactivated. This may be due to overstimulation of the M-group in an attempt to overcome an upgaze palsy. This hypothesis, which presumes that supranuclear input is reduced to the SR subnucleus but preserved to the CCN, is supported by the fact that eyelid retraction is often more prominent during attempted upgaze, when M-group excitation is expected to increase. Alternatively, eyelid retraction may be due to an underinhibition of the M-group by the iNC and nPC. Supportive of this hypothesis is the observation of lid lag (a failure of the eyelids to lower sufficiently during attempted downgaze, when inhibitory input to the M-group should be greatest) in some patients with eyelid retraction.

### Neurodegenerative Diseases Associated With Eyelid Retraction

Eyelid retraction is seen in virtually all patients with PSP (33) and is said to result in a characteristic surprised appearance or “stare.” By contrast, it has only been rarely reported in PD (34). Eyelid retraction is also a classic finding in *SCA3* (also known as *Machado-Joseph disease*); in one study, 65% of patients with SCA3 had eyelid retraction resulting in a “bulging eyes” appearance compared to less than 5% of patients with other autosomal dominant SCAs (35). Interestingly, while midbrain atrophy is the pathologic hallmark of PSP, it is rarely seen in SCA3.

### Lid Nystagmus

The close relationship between vertical eye and eyelid position applies even when eye movement is involuntary, as in upbeat nystagmus (UBN). Occasionally, rhythmic movements of the eyelids can be seen without visible UBN, resulting in the so-called eyelid nystagmus or lid flutter (36). The same mechanisms by which eye and eyelid position become dissociated in eyelid retraction are probably also responsible for the absence of eye movement in eyelid nystagmus, which is often associated with midbrain ischemic and compressive lesions (37, 38). Under normal conditions, convergence increases the basal firing rate of the LPS during primary gaze, resulting in a small degree of eyelid retraction. This may explain why eyelid nystagmus can be evoked by attempted convergence (also known as Pick’s sign) (39).

## Decreased Blinking

### Overview of Eyelid Closure

The primary muscle of eyelid closure is the OO, which is innervated by the facial nerve. It originates from multiple bony and connective tissue structures surrounding the medial canthus. The palpebral portion of this muscle—which can be further subdivided into pretarsal and preseptal components—is contained within the upper and lower eyelids and inserts on connective tissue structures surrounding the lateral canthus. The orbital portion of the OO lies outside the eyelids and forms a muscular ellipse encircling the orbit (1). The palpebral and orbital portions of the OO are innervated by separate populations of motor neurons within the facial nucleus (40). Other muscles of facial expression, such as the corrugator (which draws the eyebrows inferiorly and medially as in frowning), can secondarily contribute to eyelid closure as well (Figure 1).

### Supranuclear Control of Spontaneous Blinking

Normal spontaneous blinking occurs at a rate of 15–20 blinks/min; this frequency varies considerably between individuals and is somewhat higher in women than men (41). During a blink, the LPS abruptly stops firing, and the palpebral portion (but not the orbital portion) of the OO contracts, resulting in active eyelid closure (in contrast to the passive eyelid depression that occurs during downgaze). As soon as eyelid closure is complete, the OO abruptly stops firing, basal activity of the LPS resumes and the eyelid opens (42). The duration of eyelid closure in blinking must be very brief to avoid disrupting visual input. Blinking can also occur reflexively in response to various stimuli, including visual threat, bright light, tactile stimulation of the cornea or eyelids, and loud noise. With the exception of visual threat, which involves the occipital cortex, all are mediated by brainstem reflex arcs.

The anatomic pathways through which LPS and OO function is coordinated during blinking remains poorly understood, but the SC is thought to play a key role. The SC sends projections to both the facial motor nucleus and the supraoculomotor area directly overlying the CCN (43). It also receives afferent input from the trigeminal sensory nucleus (important for corneal and other trigeminally mediated blink reflexes) and dorsal midbrain (where reflexive blinking to light is mediated). Inhibitory microstimulation of the SC in primates has been shown to both suppress spontaneous blinking and increase sensitivity to blink reflexes (44, 45).

The SC is inhibited by the pars reticulata of the substantia nigra (SNr) through dopaminergic projections in the nigrocollicular pathway. The role of dopamine in promoting spontaneous blinking has been confirmed in animal studies showing an increase in blink rate with apomorphine and other dopamine agonists that is abolished in the presence of sulpiride, a dopamine receptor antagonist (46), and a correlation between dopamine level in the caudate nucleus and blink rate in animal models of MPTP-induced parkinsonism (47). Anticholinergics also increase blink rate, consistent with the hypothesis of dopamine–acetylcholine balance in the basal ganglia and the mechanistic rationale for anticholinergics in the treatment of parkinsonism (45).

### Reduced Spontaneous Blinking in Parkinsonism

In *PD*, the spontaneous blink rate has been found to be roughly 30% lower than healthy controls across several studies (48, 49). Blink rate increases with both levodopa therapy (50) and deep brain stimulation (DBS) of the subthalamic nucleus (51). Rarely, PD patients may have an increased spontaneous blink rate that paradoxically decreases to normal with levodopa therapy. Typically seen in cases of advanced disease, this has been postulated by some to represent a type of “off-dystonia” (52). Blink rate is dramatically reduced in PSP to as low as 3 blinks/min, making this a feature that can help distinguish it from PD. Moreover, eyelid movements themselves are of normal amplitude and velocity in PD and are, thus, not technically bradykinetic (53); by contrast, “slow blinks” have been observed in PSP (33).

Since a major function of spontaneous blinking is to evenly distribute the tear film, reduced spontaneous blinking is associated with both subjective and objective complaints of dry eye. In one study, 63% of PD patients complained of dry eye and related symptoms, and roughly 50% had objective evidence of xerophthalmia as measured by the Schirmer or tear film build-up time tests (47). Other studies have found a significantly increased prevalence of blepharitis and meibomian gland disease in PD (54). However, these findings are confounded by autonomic dysfunction in PD and may not be solely attributable to decreased blinking and inadequate tear production. Artificial tears are often recommended, but their efficacy has not been specifically studied in this population. A trial of LipiFlow (a pulsating thermal eyepiece) compared to warm compresses for the treatment of meibomian gland dysfunction in PD is currently underway (NCT02894658) (55).

### Reflexive Blinking in Parkinsonism

Given the inverse relationship between spontaneous blinking and blink reflexes, a decrease in dopamine in the SNr would be expected to enhance reflexive blinking. This manifests clinically as the glabellar reflex (trigeminally mediated blinking in response to tapping the nasion or forehead that fails to habituate, also known as Myerson’s sign), which is often present in PD (56). However, the glabellar reflex is not unique to PD and is present in many other structural, metabolic, and degenerative disorders. Reflexive blinking to other stimuli such as bright light is also increased in parkinsonism, particularly in PSP, where a lack of habituation to flashing light has been found to distinguish it from PD (57).

Reflexive blinking can be studied electrophysiologically by stimulating the supraorbital nerve and recording OO activity using surface or needle electrodes (58). This elicits two responses: R1, a brief unilateral response that occurs ipsilateral to the side of stimulation with a latency of about 10 ms, and R2, a more sustained bilateral response that occurs with a latency of about 30 ms. When the LPS is recorded, there are two corresponding periods of electromyographic silence (SP1 and SP2) such that LPS and OO activity never overlap. In addition, repetitive stimulation can be performed to assess reflex excitability. This is based on the concept of prepulse inhibition—that is, through a combination of membrane refractoriness after hyperpolarization and activation of negative feedback circuits, a second stimulus elicits a weaker response compared to the first stimulus. In the case of the blink reflex, R2 is absent when the interstimulus interval is less than 200 ms, reduced by 50–60% at an interval of 500 ms, and reduced by 10–30% at an interval of 1,500 ms.

In PD patients, the R2 latency is mildly prolonged, consistent with intrinsic brainstem pathology in the early Braak stages of the disease. R2 prolongation has been shown to be greater in PD patients with dyskinesias compared to those without and in dementia with Lewy bodies compared to PD, both of which are likely a reflection of greater Lewy body burden (59, 60). More importantly, the blink reflex is hyperexcitable in PD. In one study, for example, when the supraorbital nerve was stimulated twice over a period of 250 ms, the second R2 was 84% smaller in amplitude and 50% shorter in duration compared to the first R2 among healthy controls. By contrast, PD patients off therapy had only a 60% smaller and 10% shorter second R2 response (44). Dopaminergic therapy and STN DBS in both humans (61) and animals (62) restores blink reflex excitability to normal levels. The degree of blink reflex hyperexcitability also correlates with severity of bradykinesia, rigidity, gait impairment, dysarthria, and reduced quality of life (63, 64).

## Increased Blinking and Blink-Assisted Saccades

### Spontaneous and Reflexive Blinking in Hyperkinetic Movement Disorders

If a hypodopaminergic state reduces spontaneous blinking and increases reflexive blinking, then a hyperdopaminergic state would be expected to increase spontaneous blinking and reduce reflexive blinking. This is indeed seen in hyperkinetic movement disorders, such as *HD*. The mean blink rate in HD patients is approximately 36 blinks/min (65), nearly double the normal rate, and up to 75% of HD patients have subjectively elevated blink rates (66). In one case of juvenile HD, excessive blinking (40 blinks/min) preceded the development of other disease manifestations by over 2 years (67). Increased spontaneous blinking is the first clinical manifestation of blepharospasm (see below) and has also been described in Wilson’s disease (68). Other disorders that are thought to involve a relative excess of dopaminergic transmission and are often treated with dopamine-blocking agents, such as Tourette syndrome (69) and schizophrenia, have increased spontaneous blinking as well. The inverse relationship between spontaneous and reflexive blinking holds true in HD, as the electrophysiologic blink reflex has been shown to be underexcitable compared to normal in both symptomatic (70) and pre-symptomatic (71) individuals.

### Relationship between Blinking and Saccades

In normal individuals, spontaneous blinks are inhibited during voluntary saccades, primarily to avoid disrupting visual input during a visually guided task. Saccades are also slower and less accurate when they are interrupted by blinks (72). However, patients with parkinsonian disorders fail to suppress blinks during voluntary saccades. In one study, normal individuals blinked an average of 15.7/min when fixating in primary gaze and 9.1/min when asked to alternate looking left and right every 5 s. By contrast, PD patients experienced a slight increase in blink rate (from 12.5 to 14.8/min), and PSP patients a substantial increase in blink rate (from 3.0 to 5.3/min) during horizontal eye movements (49). The mechanism underlying this phenomenon is not entirely known, but given that spontaneous blink rates also decrease during mental tasks requiring intense concentration, the frontal lobes are thought to play a role.

While blinks reduce the speed and accuracy of saccades, they can also be used to assist saccades in disorders of saccade initiation (also known as saccadic or ocular motor apraxia). In HD, where difficulty with saccade initiation and prolonged saccadic latency are among the earliest clinical manifestations (73, 74), the use of head thrusts and blinks is initially suppressible, but as the disease progresses, patients may be unable to initiate voluntary saccades without an obligatory blink (35% in one study) (75). Blink-assisted saccades [also termed blink-saccade synkinesis (76)] are observed in other causes of impaired saccade initiation such as the autosomal dominant SCAs, the autosomal recessive ataxias with ocular motor apraxia, ataxia-telangiectasia, congenital ocular motor apraxia, Gaucher disease, Niemann–Pick disease type C, Joubert syndrome, and others (77). During normal saccade initiation, omnipause neurons in the dorsal pons cease firing, which disinhibits excitatory burst neurons in the parapontine reticular formation (for horizontal saccades) and riMLF (for vertical and torsional saccades), resulting in a saccade. Interestingly, omnipause neuron activity is also suspended during blinks (78). If ocular motor apraxia is caused by a lack of normal supranuclear inhibition of omnipause neurons during saccade initiation, then blink-saccade synkinesis may represent an alternative method of inhibiting omnipause neurons in order to generate saccades.

## Blepharospasm

### Introduction to Blepharospasm

*Blepharospasm* is characterized by periods of involuntary, sustained, forceful eyelid closure. As it involves the co-contraction of agonist (OO) and antagonist (LPS) muscles affecting eyelid position, blepharospasm qualifies as a type of focal dystonia, and more than half of patients have a *geste antagoniste* or sensory trick that can temporarily relieve symptoms (79). It typically presents between the fourth and sixth decades of life and is more common in women than men (80). The initial clinical manifestation of blepharospasm is an increase in spontaneous blink rate that paradoxically decreases with psychomotor activation (81). Over time, blinks become increasingly forceful and prolonged, involving both the orbital and palpebral portions of the OO (sometimes termed “dystonic blinks”). Eventually, these blinks coalesce into periods of sustained eyelid closure whose frequency and duration can be so severe as to produce functional blindness (82).

Blepharospasm should not be confused with eyelid myotonia, which is characterized by impaired relaxation following voluntary or reflexive (e.g., sneezing) eyelid closure. Myotonia is seen in myotonic dystrophy as well as the non-dystrophic myotonias (e.g., myotonia congenita, paramyotonia congenita), which are caused by mutations in voltage-gated chloride and sodium channel genes and are treated with sodium channel-blocking antiepileptic drugs and the antiarrhythmic drug mexilitene (83).

### Pathophysiology of Blepharospasm

It is postulated that blepharospasm represents overactivity of reflexive blinking, especially to light. Evidence for this theory comes from several observations:

1. Photophobia is an almost universal complaint in blepharospasm (84). Sun exposure has even been postulated to be a risk factor for blepharospasm given that the ratio of blepharospasm to cervical dystonia patients in movement disorders cohorts varies by season and latitude (85). Many patients report that exposure to bright light triggers spasms of eyelid closure and polarized sunglasses can be a useful adjunctive treatment (86). Ocular surface symptoms and sensitivity to tactile stimulation of the cornea and eyelids, which is also a trigger for physiologic reflexive blinking, have also been reported (87).
2. The orbital portion of the OO is normally involved in reflexive blinking but not spontaneous blinking. Given that the dystonic blinks of blepharospasm involve the orbital portion of the OO, it is suggested that they are generated *via* reflexive rather than spontaneous blinking pathways.
3. Subclinical overlap in LPS and OO activity is seen at the electromyographic level in normal reflexive blinking to light (88) but not to other stimuli. Thus, the co-contraction of these muscles in blepharospasm may represent an exaggeration of normal reflexive blinking to light.
4. The electrophysiologic blink reflex has been found to be hyperexcitable in patients with blepharospasm (89, 90). Interestingly, similar findings have been reported in patients with other focal dystonias besides blepharospasm (e.g., cervical dystonia, spasmodic dysphonia), suggesting shared pathophysiologic mechanisms (91, 92). During a *geste antagoniste*, the R2 duration shortens, but the degree of excitability does not change (93). The use of high-frequency supraorbital nerve stimulation to induce long-term depression of this reflex has been studied as a potential treatment for blepharospasm, albeit with limited success (94).

The site of pathology in blepharospasm remains unknown. The vast majority of patients have normal neuroimaging; in a single case series of 1,114 patients, only 18 (1.6%) had abnormal brain MRIs, and lesions localized to a variety of areas, including the basal ganglia, thalami, cerebellum, midbrain, and even cortex (95). Voxel-based morphometric and diffusion-tensor imaging studies have reported changes in the gray matter volume of the caudate, putamen, thalami, and cerebellum, but they have not been consistent (some reported increases, whereas others reported decreases), and it is unclear if they represent the primary site of pathology or adaptive changes in response to disease processes occurring elsewhere (96). Functional neuroimaging studies have shown hypermetabolism of a variety of cortical and deep gray matter foci, both at rest (97) and during tasks such as voluntary blinking (98), and decreased striatal dopamine binding in roughly one-third of patients (99). A case report of craniocervical blepharospasm treated with DBS found increased firing rates in the globus pallidus interna (100).

### Epidemiology and Natural History of Blepharospasm

Blepharospasm may remain limited to the OO or may spread to adjacent muscles of the face, jaw, and neck, resulting in craniocervical dystonia (also known as Meige syndrome). This spread usually occurs within 5 years of blepharospasm onset (101, 102). The lifetime risk of generalization to craniocervical dystonia has been reported to be as high as 60%, though the evidence for this comes from cohort studies recruited from tertiary movement disorders centers, where the study population may not be representative of all patients with blepharospasm (103, 104). Greater age of onset, female sex, and a prior history of minor head trauma have been identified as risk factors (105). A single nucleotide polymorphism in the 3′ untranslated region of the TOR1A gene, which is the causative gene in DYT1, was also associated with a twofold increase in risk of generalization in two separate cohort studies (106). Up to 12% of patients with blepharospasm experience spontaneous remission (107). Blepharospasm may also occur in patients with parkinsonian disorders, especially PSP but occasionally multiple systems atrophy (MSA) and rarely PD. The risk of developing PSP in patients who present with blepharospasm has not been established but is likely to be low given the rarity of the condition. However, the prevalence of blepharospasm in patients with PSP is high (anywhere from 20 to 70%) (33, 108, 109). Anywhere from 5 to 30% (110) of patients with blepharospasm also have apraxia of eyelid opening (AEO) (see below); the combination of the two is especially common in PSP. Blepharospasm has also been reported in patients with autosomal dominant SCAs (6) and neurodegeneration with brain iron accumulation (111). It can be the presenting symptom of an inherited generalized dystonia (e.g., DYT1) and may be the only clinical manifestation in families with autosomal dominant focal dystonia.

### Treatment of Blepharospasm

The treatment of choice for blepharospasm is chemodenervation of the OO with botulinum toxin. In addition to relieving the clinical symptoms of blepharospasm, botulinum toxin lowers the spontaneous blink rate (112) and reduces blink reflex hyperexcitability (113), presumably by reducing corneal sensory input from eye closure. In mild cases that present primarily with photophobia and increased blink frequency, polarized lenses may be useful. Some patients also report symptom improvement by wearing a tight band around the forehead, providing a constant *geste antagoniste*. Other conservative treatments that have been studied include behavioral therapy to encourage eyelid relaxation, biofeedback using EMG recording of the frontalis muscle (114), and transcranial magnetic stimulation (115). Medications that are typically used to treat other forms of dystonia (e.g., anticholinergics, baclofen, and benzodiazepines, levodopa) have been tried with mixed results. Prior to the advent of botulinum chemodenervation, surgical myectomy was routinely performed but is now reserved for the rare botulinum-resistant or intolerant patient (116). DBS has been performed in a few refractory cases with encouraging results (117).

## Apraxia of Eyelid Opening and Closure

### Overview of Voluntary Eyelid Control

The supranuclear control of voluntary eyelid elevation and depression is complex and poorly understood. Electrical stimulation of a number of frontal, temporal, parietal, and occipital lobar sites can elicit eye opening or closure, and the cortical control of eyelid position is thought to have a right hemispheric predominance (1). The impairment of voluntary eyelid motor control results in difficulty initiating voluntary eye opening and difficulty maintaining eye opening during the normal waking state. This syndrome was first described by Goldstein and Cogan in 1965, who called it “apraxia of lid opening” (118), though the use of the term apraxia has since been criticized, and other names, such as blepharocolysis, akinesia of lid opening and function, eyelid freezing, and involuntary levator palpebrae inhibition of supranuclear origin, have been proposed. Clinically, AEO consists of difficulty initiating eyelid elevation following sustained voluntary eyelid closure without evidence of involuntary OO activity. Electrophysiologically, it is characterized by the absence of LPS activity during attempted eyelid opening without concurrent palpebral or orbital OO activity as would be seen in light or forced voluntary eyelid closure, respectively. The frontalis muscle is often tonically activated in an attempt to secondarily elevate the upper eyelids. Spontaneous blinking and reflexive blinking are clinically and electrophysiologically normal, confirming that the neuromuscular apparatus of the levator palpebrae is intact and that the disorder is one of supranuclear control. PET studies have demonstrated medial frontal hypometabolism in the anterior cingulate and supplemental motor areas (119, 120).

There is evidence to suggest that at least a subset of patients with AEO may have a form of dystonia. As many as one-third of patients with AEO report a *geste antagoniste*, typically a light touch of the eyelids, that allows for temporary eye opening (121). Blepharospasm may co-exist, and AEO is occasionally unmasked by chemodenervation of the OO to treat blepharospasm, mistaken as treatment failure or ptosis due to the spread of botulinum toxin and treated with the addition of botulinum toxin to the pretarsal OO (122–124). While the OO is by definition clinically and electrophysiologically silent in AEO, selective electromyographic recordings of the pretarsal portion of the OO have revealed the presence of abnormal activity in some patients (125). Because of its technical challenges, this finding has been difficult to replicate on a larger scale, and it is unclear if these patients truly have AEO, a subtle variant of blepharospasm, or a distinct entity altogether that some have termed “OO motor persistence.”

### Neurodegenerative Diseases Associated With AEO

Apraxia of eyelid opening may occur in isolation or in association with an underlying neurodegenerative disorder. Of 32 patients with AEO seen at a regional referral center in Puglia, Italy, over a 10-year period, 10 were healthy, 10 had blepharospasm, 6 had PSP, and 3 had idiopathic PD (126). The number of patients with AEO who have PD may be similar to the number of patients who have PSP, but because PSP is so much rarer than idiopathic PD, the prevalence of AEO is much higher in PSP. Anywhere from 30 to 45% of patients with PSP experience AEO (127). Furthermore, AEO typically coincides with or precedes the onset of parkinsonism in PSP, whereas it is a much later manifestation of PD. AEO is also seen in MSA and corticobasal syndrome, though in the latter the underlying pathology at autopsy is one of PSP rather than true corticobasal ganglionic degeneration (128). It has also been described in cases of amyotrophic lateral sclerosis (ALS) with or without frontotemporal disease, HD, SCA2 (129), SCA3 (130), Wilson’s disease, chorea-acanthocytosis, and others.

A benign unilateral AEO has been described which typically occurs on awakening and resolves after manual elevation of the affected eyelid (131). While this could represent a *geste antagoniste*, EMG studies are lacking due to the transient nature of these symptoms, and it is unclear whether this condition reflects excess OO activation or excess LPS inhibition (132). Apraxia of eyelid *closure* has also been described but is much less common. These patients constrict the corrugator and procerus muscles during attempted voluntary eyelid closure but not the OO (133); however, they are able to close their eyes normally during spontaneous and reflexive blinking. It has been reported in patients with PSP (33), HD (134), Creutzfeldt–Jakob disease, ALS (135), and acquired frontal and parietal lobe disease.

### Treatment of AEO

The treatment of AEO requires a multimodal approach. Conservative measures include wearing goggles (136) or eyelid crutches (137); these serve to mechanically elevate the upper eyelid but likely also act as a *geste antagoniste*. Levodopa may improve AEO when it is isolated (138) or associated with PD (139, 140) but appears to worsen it when associated with PSP. Other medications that have been tried on a case-by-case basis include anticholinergics (141), atypical antipsychotics (142), and methylphenidate (143). Given the finding of abnormal EMG activity in the pretarsal OO in some patients with AEO, botulinum injection of the pretarsal OO is frequently performed with success (144). In cases of comorbid blepharospasm and AEO, surgical myectomy (145) or frontalis suspension (146, 147) can treat both disorders simultaneously but are generally reserved as a last resort.

The association between AEO and PD deserves special attention as it can be confounded by DBS (148). While AEO may be present in untreated PD, it can emerge or worsen after STN DBS (or posteroventral pallidotomy during the pre-DBS era) in anywhere from 2 to 31% of patients, presumably *via* the spread of current into the adjacent corticobulbar tract, particularly when higher voltages are applied to more caudal contact points. In fact, experimental low-frequency stimulation of the STN at certain voltage thresholds has been shown to induce myoclonus in the pretarsal OO (149). The weaning of levodopa following DBS may also unmask pre-existing symptoms (150). AEO usually occurs within a year of DBS implantation. An increase in spontaneous blink rate can be a harbinger of AEO during programming sessions (151). Treatment is challenging and consists of reducing voltage, increasing frequency, and administering levodopa in addition to conventional therapies. Paradoxically, some patients with AEO experience improvement with STN (151, 152) or GPi (153) DBS.

## Author Contributions

AH performed the primary literature review and drafted the manuscript. DG conceived of the manuscript idea and edited the manuscript.

## Conflict of Interest Statement

The authors declare that the research was conducted in the absence of any commercial or financial relationships that could be construed as a potential conflict of interest.
